# Service Benefit Aware Multi-Task Assignment Strategy for Mobile Crowd Sensing

**DOI:** 10.3390/s19214666

**Published:** 2019-10-27

**Authors:** Zhidu Li, Hailiang Liu, Ruyan Wang

**Affiliations:** 1 School of Communication and Information Engineering, Chongqing University of Posts and Telecommunications, Chongqing 400065, China; lizd@cqupt.edu.cn (Z.L.); wangry@cqupt.edu.cn (R.W.); 2 Key Laboratory of Optical Communication and Networks, Chongqing 400065, China; 3 Key Laboratory of Ubiquitous Sensing and Networking, Chongqing 400065, China

**Keywords:** Mobile Crowd Sensing, task assignment, service benefit, users clustering, gradient descent algorithm

## Abstract

Mobile crowd sensing (MCS) systems usually attract numerous participants with widely varying sensing costs and interest preferences to perform tasks, where accurate task assignment plays an indispensable role and also faces many challenges (e.g., how to simplify the complicated task assignment process and improve matching accuracy between tasks and participants, while guaranteeing submitted data credibility). To overcome these challenges, we propose a service benefit aware multi-task assignment (SBAMA) strategy in this paper. Firstly, service benefits of participants are modeled based on their task difficulty, task history, sensing capacity, and sensing positivity to meet differentiated requirements of various task types. Subsequently, users are then clustered by enhanced fuzzy clustering method. Finally, a gradient descent algorithm is designed to match task types to participants achieving the maximum service benefit. Simulation results verify that the proposed task assignment strategy not only effectively reduces matching complexity but also improves task completion rate.

## 1. Introduction

The diversification and popularization of embedded mobile devices enable innumerable user-centric mobile crowd sensing (MCS) applications (e.g., traffic monitoring, pollution monitoring, and indoor positioning) [1,2,3,4,5,6,7,8,9]. A typical MCS system includes two entities (i.e., users and platform), where users not only publish tasks to acquire information from the platform but also collect sensing data for the platform. As a bridge between task publishers and participants, the platform helpfully selects suitable participants to complete tasks for publishers. Generally, sensing tasks in a MCS system are allocated to multiple participants and accomplished cooperatively [10,11,12,13,14].

It is crucial for a MCS system to provide task publishers with reliable services. The task sensing process of MCS system relies on massive participants whose sensing positivity and sensing capacity are diverse for different tasks. If a task is randomly assigned to participants, the quality of its results may be severely affected, the credibility of collected sensing data may be reduced, and the corresponding computing resource consumption may increase [15,16,17,18,19,20]. Therefore, task assignment strategies should be reasonably designed to eliminate the above uncertainties, which can dramatically enhance the philosophy behind MCS participant collaborations [21,22,23].

Technically, how to objectively evaluate the matching accuracy of task assignment strategies is a major challenge and remains in dispute. In response to this challenge, many studies presented solutions using varying methods and with different emphases. Jin, H et al. assigned MCS tasks to participants according to their capabilities to maximize sensing coverage [24]. The authors of [25,26] assigned tasks based on the history of submitted high-quality results. However, they had different definitions of data quality. Yue, W et al. mainly considered the coverage quality of sensing results [25], while Sabrina, K.N.M et al. measured the data quality based on the actual evaluation of data [26]. Liu, S et al. evaluated participant’s service quality based on its context and cost, and developed a Modified Thompson Sampling Worker Selection (MTS-WS) algorithm to select workers in a reinforcement learning manner [27]. Addressing the problem of performance maximization in MCS, a context-aware hierarchical online learning algorithm was proposed in [28]. In detail, a local controller (LC) in the mobile device of a participant regularly observed the participant’s context, based on which the participant’s context-specific performance could be estimated and the participants could be selected. Although the authors of [24,25,26,27,28] considered multiple task-related factors, there are still some limitations and factors that may intuitively affect MCS performance (e.g., task difficulty, task history, sensing capacity, and sensing positivity were omitted). Besides, the quality of data uploaded for different task types was not clearly modeled in these studies. Consequently, it may be impossible for the platform to assign tasks reasonably and accurately, resulting in low matching accuracy/task completion rate, high computing resource consumption, and incredibility of data.

Focusing on above limitations, we propose a service benefit aware (SBAMA) multi-task assignment strategy for MCS. The service benefit of participants is first modeled. Subsequently, we propose an enhanced Fuzzy C-Means (FCM) algorithm to dynamically cluster users in terms of their task preferences. Finally, an iterative participant search method based on gradient descent is designed to match participants with the best service benefit in each cluster quickly and accurately. The proposed SBAMA multi-task assignment strategy offers an interest tradeoff between participants and platform given a fixed budget and a certain movement distance. Simulation results verify that SBAMA can quickly and accurately find the most appropriate participants for all types of tasks. Therefore, task completion rate based on the proposed strategy is convincingly high. The main contributions of our paper can be summarized as follows:

(1) A service benefit evaluation model is established, from several different perspectives of tasks and participants, to comprehensively interpret impacts of task difficulty, task history, sensing capacity, and sensing positivity on service benefit received by the platform.
(2) An enhanced FCM algorithm is designed to cluster users. Specifically, a task preference threshold allows participants to join more than one cluster if such participants have similar task preferences. The generated clusters can effectively reduce the time consumption of the optimization problem while increasing participant matching accuracy.
(3) An iterative gradient descent algorithm is proposed to tune the tradeoff between interests of participants and platform. Particularly, it decouples the service benefit from movement distance such that the most appropriate participants for tasks can be found accurately and quickly.

The rest of this paper is organized as follows. Related works are introduced in Section 2. Section 3 presents the task assignment framework. The service benefit model is proposed in Section 4. Section 5 elaborates on the task assignment strategy. Simulation results validating our proposed SBAMA are given in Section 6. Finally, Section 7 concludes this paper and discusses future work.

## 2. Related Works

In recent years, task assignment for MCS systems has been attracting increasing research attention. Wei, G et al. proposed a heterogeneous multi-task allocation mechanism based on spatiotemporal correlation [29]. The reference and non-reference tasks were distinguished through utilizing granularity settings based on which the best triple (i.e., worker-cycle-region) was obtained. Besides, to improve the task assignment efficiency, a decomposition and combination framework was designed in [29] for large-scale scenarios. In [30], a location-based online task assignment method was proposed under constraints of distance and budget, incorporating quality/progress-based, task-density-based, travel-distance-balance-based, and bio-inspired-travel-distance-balance-based algorithms, to search for the optimal participants maximizing the overall task quality. Wang, J et al. studied the deterministic model and random model of trajectories in vehicle-based MCS and proposed an effective vehicle recruitment algorithm to minimize the overall recruitment cost [31]. However, the authors of [29,30,31] only considered either the mobility of participants or the spatiotemporal correlation among tasks, where impacts of task requirements and service benefits were ignored. A failure to consider these critical factors together may result in an inaccurate participant matching during the task assignment process, which reduces the task completion rate.

The criterion of participant selection in task assignment has been extensively studied. Considering various factors affecting the task participant selection, a task assignment framework was proposed in [32]. Specifically, a unified estimation function was employed to calculate the feasibility of task assignments and the optimal task assignment using a greedy algorithm was obtained. Wang, L et al. and Alsayasneh, M et al. focused on the context information of participants to enhance the task completion rate and MCS quality [28,33]. In particular, a diverse task composition scheme was studied in terms of participant personalities to dramatically improve user experience [33]. Mavridis, P et al. inferred skills required for tasks from available skill sets and modeled a hierarchical skill tree to match participants with tasks, which was however computationally intensive and therefore inapplicable to scenarios with massive users and tasks [34]. Besides, the credibility of data submitted by participants could not be guaranteed only based on their skills. Although the authors of [28,32,33,34] assigned tasks to relatively appropriate participants to ensure the data credibility, when evaluating the participant selection, key factors such as participant positivity and task difficulty should be carefully considered for the matching accuracy. Therefore, in this paper, we comprehensively evaluate the service benefits of participants as the matching criterion and propose the SBAMA multi-task assignment strategy to enhance MCS performance.

## 3. Task Assignment Framework

**Figure 1:**
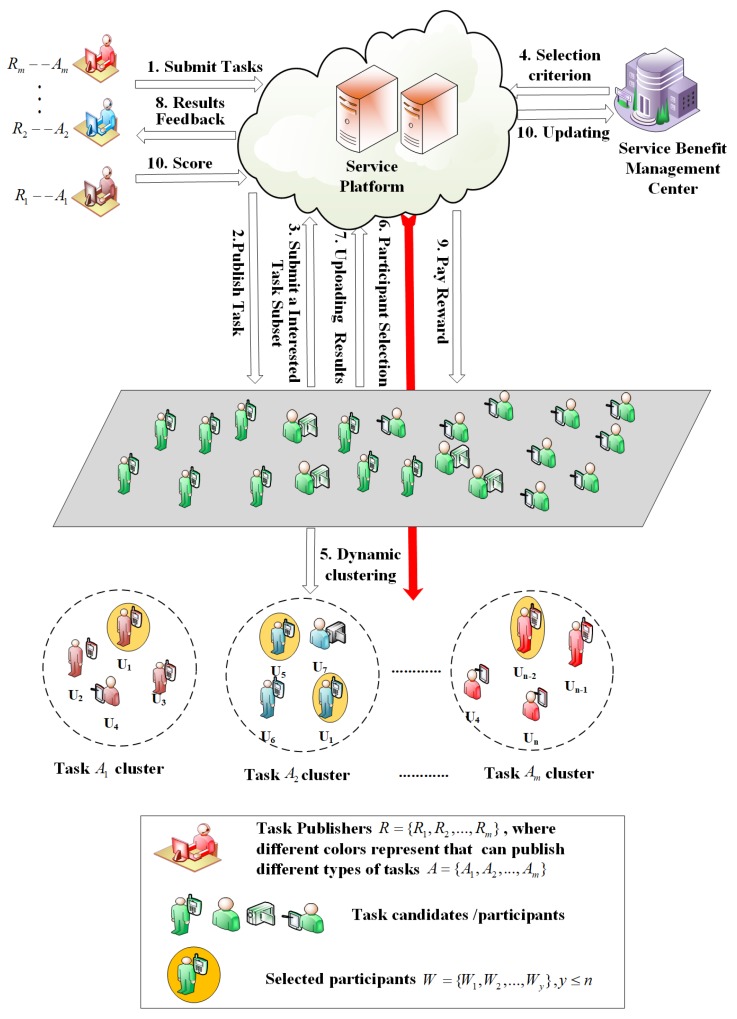
SBAMA framework.

The proposed SBAMA framework is shown in Figure 1. Since tasks arrive randomly, tasks assigned by the platform can be divided into *n* identical time intervals, denoted as $t=[t_{1},t_{2}\ldots \ldots t_{\eta }]$. At the beginning of $t_{\eta }$, task publishers $R=\left\{R_{1},R_{2}\ldots \ldots R_{m}\right\}$ submit tasks of different types $A=\left\{A_{1},A_{2}\ldots \ldots A_{m}\right\}$ to the platform. Each publisher can only submit one type of tasks within each time interval. Note that each task type contains *q* subtasks, i.e., $A_{i}=\left[a_{1}^{i},a_{2}^{i}\ldots \ldots a_{q}^{i}\right]$. Specifically, a task has several requirements (i.e., deadline, location, data format, and ID), denoted as $T_{doc}=\langle T_{d};l_{A_{i}};\varpi ;ID\rangle$, where $T_{d}$ refers to the time range from task start $t_{s}$ to task end $t_{e}$ (i.e., $T_{d}=\left[t_{s},t_{e}\right]$) and participants must submit sensing results before this deadline. The specific locations for subtasks of task $A_{i}$ are denoted by $l_{A{\;}_{i}}=\left[l_{i1},l_{i2}\ldots \ldots l_{iq}\right]$. Due to different content types for each task, the format of collected data also varies. Without loss of generality, the data format is identified by $\varpi =\left[\varpi_{1},\varpi_{2}\ldots \ldots \varpi_{k}\right]$, where $\varpi_{k}$ specifies the content type acceptable for each task.

The platform then publishes tasks to all potential participants $U=\left\{u_{1},u_{2}\ldots \ldots u_{n}\right\}$ satisfying task requirements $T_{doc}$. Task candidates then submit a subset of the received tasks $SA=\left[A_{1},A_{2}\ldots \ldots A_{k}\right]\;,\;\;\;\mathrm{where}\;k\leq m,$ to the platform indicating their task preferences. Subsequently, the platform employs task assignment strategy to select participants with high service benefits and low costs to perform tasks. In other words, the optimal task participants $W=\left(W_{1},W_{2}.....W_{y}\right)$(y≤n) are found.

When tasks are completed, the platform compensates the selected participants according to their costs and task difficulties. Finally, upon receiving feedback from the platform, task publishers score the service quality of the participants. Apparently, the scores should be exploited by the platform as an important reference to evaluate and update the service benefit, which further serves as an indicator for the next rounds of task assignments.

Generally, MCS tasks are location-dependent and participants have to travel a certain distance to perform the tasks. Therefore, movement distances are an inevitable cost for participants, i.e., cost $C_{ij}$ of participant $u_{j}$ performing task $A_{i}$ should be a function of movement distance $d_{ij}$ and sensing cost $c_{ij}$. Evidently, $C_{ij}$ is proportional to the distances traveled by participants (i.e., $C_{ij}\left({\tilde{d}}_{ij}\right)\geq C_{ij}\left(d_{ij}\right),\forall \;\;{\tilde{d}}_{ij}\geq d_{ij}$) and its growth rate also should increase with the distance (i.e., $\frac{d^{2}C_{ij}\left(d_{ij}\right)}{dd_{ij}}\geq 0$). The cost $C_{ij}$ is defined as follows

(1) $$C_{ij}=a_{ij}d_{ij}^{2}+b_{ij}d_{ij}+c_{ij}$$

In Equation (1), $a_{ij}>0$ and $b_{ij}>0$ are pre-defined system parameters and $c_{ij}$ is constant. Participants expect to get rewards from the platform after completing tasks. Specifically, the reward depends on the task difficulty and movement distance. Given a difficult task, its price per meter should be high, and prices of different types of tasks are denoted by $P=[P_{1},P_{2}\ldots \ldots P_{m}]$. Besides, the reward should not exceed the budget. Note that participants can perform different subtasks in the same time interval to maximize their incomes, only when their locations are not in conflict. Thus, the income of participants can be easily calculated through deducting the cost from the reward; there holds

(2) $$\;\;\;\;\;\;\psi_{j}\left(d_{ij}\right)=\sum_{i=1}^{m}\left[\left(P_{i}\cdot d_{ij}\right)-C_{ij}\;\;\;\;\;\;\;\right]\;\;\;\;$$

For the platform, its profit mainly comes from service benefits contributed by participants. Intuitively, participants with greater service benefits generate more profits for the platform and therefore should be prioritized. However, the growth rate of the profit should be slowly attenuated because the participant service benefits become smaller and smaller after the participant is selected. The profit obtained from completing task can be calculated as follows

(3) $$f_{i}\left(s_{ij},d_{ij}\right)=\sum_{j=1}^{n}\left[w_{i}\cdot \ln\left(1+s_{ij}\right)\right]-\sum_{j=1}^{n}\left(P_{ij}\cdot d_{ij}\right)$$

where system coefficient $w_{i}>0$ is determined by the platform for each task type. Apparently, both the platform and participants want to maximize their profits or incomes. Hence, a reasonable task assignment strategy should select participants with low sensing costs and high service benefits, where a tradeoff between the platform and participants must also be made under constraints of the maximum movement distance $r_{j}^{\max}$ and budget of each type of tasks $B=\left[B_{1},B_{2}\ldots \ldots B_{m}\right]$. Rationally, the reward should be more than the cost to motivate participants to travel within $r_{j}^{\max}$. Eventually, the task assignment can be formulated as the following optimization problem.

(4) $$\max\;\;\;\;\;\;\psi_{j}\left(d_{ij}\right)=\sum_{i=1}^{m}\left[\left(P_{i}\cdot d_{ij}\right)-C_{ij}\;\;\;\;\;\;\;\right]\;\;\;\;$$

(5) $$\max\;\;\;\;\;f_{i}\left(s_{ij},d_{ij}\right)=\sum_{j=1}^{n}\left[w_{i}\cdot \ln\left(1+s_{ij}\right)\right]-\sum_{j=1}^{n}\left(P_{i}\cdot d_{ij}\right)\;\;\;\;\;\;\;$$

(6) $$\begin{array}{c} s.t.\;\;\;\;\;\sum_{i=1}^{m}d_{ij}\leq r_{j}^{\max},d_{ij}>0\\ \;\;\;\;\;\;\;\;\;\;\;\;\;\;\;\;\;\;\;\;\;\sum_{j=1}^{n}P_{i}\cdot \;\;d_{ij}\leq B_{i}\\ \;\;\;\;\;\;\;\;\;\;\;\;\;\;\;\;0<s_{ij}\leq 1 \end{array}$$

## 4. Service Benefit Evaluation

Service benefit is an important indicator for the platform to estimate the potential profit gained from a certain participant, which often relates to task requirements. In this paper, task difficulty, task history, sensing capacity, and sensing positivity are employed, from perspectives of tasks, participants, and publishers, to comprehensively evaluate the service benefit of all types of tasks, so as to achieve accurate participant matching and reliable MCS data collection.

### 4.1. Sensing Positivity

Sensing positivity refers to the motivation of participants in performing sensing tasks, which is a dynamic process. Given the same sensing capacity, a higher sensing positivity signifies a greater contribution to the platform. Interactions between participants and the platform are employed to measure the sensing positivity, where interaction frequency and task performance are two major observable indicators for these interactions. Specifically, performance *p* is a function of response time Δt and cost $C_{ij}$. If a participant has a relatively low Δt and $C_{ij}$, the platform deems his/her performance positive. Due to the restrictive task deadline and movement distance, *p* decreases with the growing Δt and $C_{ij}$, and then gradually stabilizes. Therefore, performance $p_{ji}$ of participant $u_{j}$ in task $A_{i}$ can be calculated by

(7) $$p_{ji}=\log_{2}\left(\frac{1}{C_{ij}\cdot \Delta t}+1\right)$$

where $\Delta t=\left|t_{q}^{i}-t_{s}^{i}\right|$, $\Delta t\leq T_{d}$. $t_{q}^{i}$ is the time participant $u_{j}$ starts task $A_{i}$ and obviously Δt=0 if $t_{q}^{i}$ is equal to task start time $t_{s}^{i}$, which generates the value of maximum performance is 1. Conversely, the minimum performance 0 can be obtained when $\Delta t=T_{d}$. Besides, when $C_{ij}$ is infinitely large, $p_{ji}$ reaches 0 (i.e., $\log_{2}1=0$), as bounded by

(8) $$p_{ji}=\left\{\begin{array}{c} 0\;\;\;\;\;\;\;\;\;\;\;\;\;\;\;\;\;\;\;\;\;\;\;\;\;\;\;\;\;\;\;\;\;\;\;\;\;\;\;\;\;\;\;\;\;\;\;\;\;\;\;\;\;\;\;,\;\;\Delta t=T_{d}\\ \log_{2}\left(\frac{1}{C_{ij}\cdot \Delta t}+1\;\right)\;\;\;\;\;\;,0<\Delta t<T_{d}\\ 1\;\;\;\;\;\;\;\;\;\;\;\;\;\;\;\;\;\;\;\;\;\;\;\;\;\;\;\;\;\;\;\;\;\;\;\;\;\;\;\;\;\;\;\;\;\;\;\;\;\;\;\;\;\;\;\;\;,\;\;\;\Delta t=0 \end{array}\right.$$

Interaction frequency is another important indicator for sensing positivity. Apparently, a high interaction frequency signifies a stable and positive sensing behavior, and thereby the interaction frequency has the equivalent weight with the task performance in sensing positivity. Therefore, the sensing positivity of participant $u_{j}$ in task $A_{i}$ can be obtained as

(9) $$\chi_{j}^{i}=\frac{f_{jm}}{\sum_{j=1}^{n}\sum_{i=1}^{m}f_{ji}}\cdot p_{ji}$$

In Equation (9), $f_{j}=\left[f_{j1},f_{j2}\ldots f_{jm}\right],f_{jm}=\left[1,2\ldots \ldots h\right]$ refers to the tasks participated by $u_{j}$, and *h* denotes the latest task. The sensing positivity for different types of tasks is represented by a vector $X_{j}=\left[\chi_{j}^{1},\chi_{j}^{2},\chi_{j}^{3}\ldots \ldots \chi_{j}^{m}\right]$.

### 4.2. Task Difficulty

The task difficulty challenges the sensing capacity of participants, and we utilize a difficulty coefficient to measure it in this paper, where a small coefficient signifies a difficult task. However, dynamic MCS tasks are large in number and rich in type. Evaluating the task difficulty in real time will inevitably consume massive computing power of the platform, which is prohibitively expensive. Therefore, we employ an offline method evaluating the completion rate in task history to obtain the difficulty coefficient. Specifically, the completion rate is defined as the ratio of completed subtasks $\left|A_{competed}^{i}\right|$ to all published subtasks *q*, $\ell_{i}=\frac{\left|A_{competed}^{i}\right|}{q}$ and $\left|A_{competed}^{i}\right|\leq q$. Note that the completion rate of different types of tasks is denoted by a vector $\ell =\left[\ell_{1},\ell_{2}\ldots \ldots \ell_{m}\right]$. Intuitively, a high completion rate signifies a simple task. Besides, the completion time must be before the deadline. Here, we exploit a theoretical completion time $\vartheta =\left[\vartheta_{1},\vartheta_{2}\ldots \ldots \vartheta_{m}\right]$ to evaluate the actual completion time $\tilde{\vartheta }=\left[{\tilde{\vartheta }}_{1},{\tilde{\vartheta }}_{2}\ldots \ldots {\tilde{\vartheta }}_{m}\right]$, and they are calculated by ${\tilde{\vartheta }}_{i}=\sum_{q=1}^{\left|A_{competed}^{i}\right|}a_{q}^{i}\cdot t_{make-span}^{actual}$ and $\vartheta_{i}=q\cdot \sum_{a_{q}\in A_{i}}t_{make-span}^{theoretical}$, where *q* is the total number of published subtasks. If ${\tilde{\theta }}_{i}$ is within $\vartheta_{i}$, this task can be completed. Eventually, the difficulty coefficient can be obtained as

(10) $$D_{i}=\left\{\begin{array}{c} \ell_{i}\times \frac{\left(\vartheta_{i}-{\tilde{\vartheta }}_{i}\right)}{\max\left(\vartheta_{i}-{\tilde{\vartheta }}_{i}\right)},\vartheta_{i}\geq {\tilde{\vartheta }}_{i}\\ \ell_{i}\times \left(1-\frac{\left({\tilde{\vartheta }}_{i}-\vartheta_{i}\right)}{\max\left({\tilde{\vartheta }}_{i}-\vartheta_{i}\right)}\right),\vartheta_{i}<{\tilde{\vartheta }}_{i} \end{array}\right.,\vartheta_{i}\leq T_{d}$$

It is also worth noting that the task difficulty is relative. If the sensing capacity of a participant is low, the platform will not assign a difficult task to him/her. We denote the varying sensing capacity of participants with a vector $K=\left[\kappa_{1j},\kappa_{2j}\ldots \ldots \kappa_{ij}\right]$, where $\kappa_{ij}$ is a constant determined by hardware specifications of sensing devices. Therefore, the relative difficulty coefficient of participant $u_{j}$ performing task $A_{i}$ can be easily calculated and normalized using logarithmic function, as shown in the following

(11) $${ℑ}_{ij}=\;\left\{\begin{array}{c} \log_{2}\left(\left|\kappa_{ij}-D_{i}\right|+1\;\;\right)\;\;\;\;\;\;\;,\;\;\;\;\;\;\vartheta_{i}\geq {\tilde{\vartheta }}_{i}\\ \log_{2}\left(\left|\kappa_{ij}-D_{i}\right|\;\;+1\right)\;\;,\;\;\;\;\;\;\vartheta_{i}<{\tilde{\vartheta }}_{i} \end{array}\right.,\vartheta_{i}\leq T_{d}$$

Relative difficulty coefficients are further denoted by matrix $C_{{}_{n\times m}}$, as shown in Equation (12), where rows represent participants and columns indicate tasks.

(12) $$C_{n\times m}=\left[\begin{array}{cccc} {ℑ}_{1,1} & {ℑ}_{1,2} & \ldots \ldots & {ℑ}_{1,m}\\ {ℑ}_{2,1} & {ℑ}_{2,2} & \ldots \ldots & {ℑ}_{2,m}\\ \ldots & \ldots & \ldots \ldots & \ldots \\ {ℑ}_{n,1} & {ℑ}_{n,2} & \ldots \ldots & {ℑ}_{n,m} \end{array}\right]$$

### 4.3. Task History

Generally, subjective feedback from task publishers is an effective benchmark for the credibility of data submitted by participants. However, due to insufficient labeled MCS data, it is challenging to objectively evaluate the data credibility. We exploit historical records of participants including ID, collected data format $\varpi_{j}$, $\varpi_{j}\in \varpi$ and reward ${ℜ}_{j}$, ${ℜ}_{j}=P_{i}\cdot d_{ij}$, as denoted by $Z_{j}=\langle \#,\varpi_{j},{ℜ}_{j}\rangle$, to evaluate data credibility. Collected data format $\varpi_{j}$ is compared with task requirements ϖ defined by $T_{\mathrm{doc}}$, and a small gap signifies the complete data, which can be indicated by $I_{j}=\frac{\left|\left|\varpi \right|-\left|\varpi_{j}\right|\right|}{\max\left|\left|\varpi \right|-\left|\varpi_{j}\right|\right|}$. Besides, the more data formats the platform receives, the higher data credibility a task can obtain. Generally, the value of data is defined as a quotient of frequency and the residual of $\varpi_{j}$ in the task history of publisher $R_{i}$. The value of the historical data is estimated by linear regression model Y=Xω+δ, where *Y* and δ are both $\left|U\right|$ dimension vector, $\left|U\right|$ denotes the number of participants, *X* is a $\left|U\right|\times \left|Z_{i}\right|$ matrix, and ω is a $\left|Z_{i}\right|$ dimension vector. Therefore, the residual between the actual data value and the estimated is $\hat{\delta }=Y-\hat{Y}=\left(1-M\right)Y$, where $M=X{\left(X^{T}X\right)}^{-1}X^{T}$ is a hat matrix and a small residual indicates high data credibility. Consequently, the data credibility of participant $u_{j}$ for task $A_{i}$ can be obtained as $reliable_{ij}=\frac{I_{j}\cdot f_{value}^{j}}{∥\hat{\delta }∥}$. Moreover, a publisher gives a high score to participants requiring relatively low task payments, and the score given to participants can be obtained as follows

(13) $$n_{ij}=\frac{relible_{ij}}{{ℜ}_{j}}$$

We further adopt the logarithm to normalize the score, as shown in the following

(14) $$n_{ij}=\log_{2}\left(1+\frac{relible_{ij}}{{ℜ}_{ij}}\right)$$

Then, the score matrix $N_{n\times m}$ can thus be obtained as follows

(15) $$N=\left[\begin{array}{ccccc} n_{11} & n_{12} & n_{13} & \ldots \ldots & n_{1m}\\ n_{21} & n_{22} & n_{23} & \ldots \ldots & n_{2m}\\ n_{31} & n_{32} & n_{33} & \ldots \ldots & n_{3m}\\ \ldots & \ldots & \ldots & \ldots \ldots & \ldots \\ n_{n1} & n_{n2} & n_{n3} & \ldots \ldots & n_{nm} \end{array}\right]$$

where rows represent task publishers and columns represent participants.

### 4.4. Service Benefit

Mathematically, the service benefit of participants is a function of task score $n_{ij}$, relative task difficulty ${ℑ}_{ij}$, and sensing positivity $\chi_{j}^{i}$, where $\chi_{j}^{i}$ serves as the weight of the service benefit indicating the motivation of participants. In addition, the service benefit grows monotonically with the increasing task score and difficulty. As marginal benefits of submitted data gradually decrease, the growth rate of service benefits drops and stabilizes. Thus, the service benefit of $u_{j}$ to $A_{j}$ can be formulated through the following inverse trigonometric function

(16) $$s_{ij}=\frac{\chi_{j}^{i}}{\pi }\times \arctan\left[n_{ij}\cdot {ℑ}_{ij}\right]+\frac{1}{2}$$

However, the evaluation of service benefits depends on task history, which is inapplicable to new participants. Therefore, we propose to set the default value of service benefits to 0.5, indicating an uncertain service benefit for strange participants. Besides, 0.5 also serves as a threshold to distinguish participants with low service benefits. Based on their task histories, we can reformulate the service benefit of participants as

(17) $$s_{ij}=\left\{\begin{array}{c} {}_{\;}^{\frac{\chi_{j}^{i}}{\pi }\times \arctan\left[n_{ij}{ℑ}_{ij}\right]+\frac{1}{2},\;\;\;\;\;\;\;\;task\;\;history}\\ 0.5\;\;\;\;\;\;\;\;\;\;\;\;\;\;\;\;\;\;\;\;\;\;\;\;\;\;\;\;\;\;\;\;\;\;\;\;\;\;\;\;\;\;\;\;\;\;\;\;\;\;\;\;\;,no\;\;task\;\;history \end{array}\right.$$

Similarly, service benefits of participants for different types of tasks can also be denoted by matrix $S_{n\times m}$.

## 5. Service Benefit Aware Multi-Task Assignment

Technically, the optimization goal of MCS task assignment is to select participants with high service benefits and low costs, so as to balance the interests of participants and the platform, given constraints of movement distance and budget. To solve this optimization problem, we first cluster users (i.e., task candidates) according to their task preferences, and then exploit a gradient descent algorithm to find the optimal participants in each cluster.

### 5.1. User Clustering Based on Task Preference

In MCS scenarios with massive users, the matching accuracy of optimization algorithms always suffers from the large search range of task candidates. Therefore, we propose to employ the similarity among task preferences to cluster task candidates. Specifically, task preferences indicate the interest of users in certain tasks, which can be reflected by task acceptance rate and task performance. The task acceptance rate is defined as the proportion of tasks submitted by user $u_{j}$ to the total number of tasks submitted by all the selected participants, calculated by $p_{acc}^{jm}=\frac{{\left|SA_{i}\right|}_{u_{j}}}{\sum_{u_{j}\in W}\sum_{i=1}^{k}{\left|SA_{i}\right|}_{u_{j}}}$, which further serves as the weight of the task preference. Intuitively, the acceptance rate of participant, calculated by $p_{acc}^{jm}=\frac{{\left|SA_{i}\right|}_{u_{j}}}{\sum_{u_{j}\in W}\sum_{i=1}^{k}{\left|SA_{i}\right|}_{u_{j}}}$, which further serves as the weight of the task preference. Intuitively, the acceptance rate of participant $u_{j}$ for different types of tasks can be denoted by vector $P_{acc}^{ji}=\left[p_{acc}^{j1},p_{acc}^{j2}\ldots \ldots p_{acc}^{jm}\right]$. The task preferences of users $p_{j}=\left[p_{j1},p_{j2}\ldots \ldots p_{jm}\right]$ are also perceived by the platform, which can be calculated similarly with Equation (8). Therefore, task preferences can be denoted by the product of the task acceptance rate and task performance (i.e., $h_{ij}=p_{acc}^{ji}\times p_{ji}$ ), and its matrix holds as

(18) $$H=\left[\begin{array}{ccccc} h_{11} & h_{12} & h_{13} & \ldots \ldots & h_{1m}\\ h_{21} & h_{22} & h_{23} & \ldots \ldots & h_{2m}\\ \ldots & \ldots & \ldots & \ldots \ldots & \ldots \\ h_{n1} & h_{n2} & h_{n3} & \ldots \ldots & h_{nm} \end{array}\right]$$

where rows represent users and columns represent tasks.

The number of MCS clusters depends on the number of published tasks in each time interval, which varies dynamically with the task preference. Note that a user may be interested in multiple tasks and therefore belongs to more than one cluster, which makes Fuzzy C-Means (FCM) algorithm a perfect clustering method for this scenario. In terms of the task preference defined above, we employ cosine similarity to replace the Euclidean distance in standard FCM and modify it into similarity FCM (SFCM). The cosine similarity of task preferences indicating the preference similarity between cluster center $o_{k}$ and user $u_{j}$ in SFCM can be calculated by

(19) $$d_{kj}=1-\cos\left(h_{k},h_{j}\right)=1-\frac{\sum_{i=1}^{m}h_{ji}\times h_{ki}}{\sqrt{\sum_{i=1}^{m}h_{ji}^{2}}\sqrt{\sum_{i=1}^{m}h_{ki}^{2}}}$$

In FCM, the fuzzy weighted exponent *m* is commonly employed to determine the fuzzy degree of clustering results, and its optimal value is usually set to 1.5≤m≤2.5. We take m=2 and the objective function of SFCM can be obtained as follows

(20) $$\begin{array}{c} \;\;\;\;\;\;\;\;\;\;\;J\left(U,O\right)=\sum_{j=1}^{n}\sum_{k=1}^{\Lambda }{\left(\mu_{kj}\right)}^{2}{\left(d_{kj}\right)}^{2}\\ s.t.\;\;\;\;\left\{\begin{array}{c} 0\leq \mu_{kj}\leq 1\;\;\;\;\;\;,k\in \left[1,\Lambda \right],j\in \left[1,n\right]\\ \sum_{k=1}^{\Lambda }\mu_{kj}=1\;\;\;\;\;\;\;\;\;\;\;\;\;\;\;\;\;\;\;\;\;\;\;\;\;\;\;\;\;\;\;\;\;\;\;\;\;\;\;\;\;\;\;\;\;\;\;\;\;\;\;\;,j\in \left[1,n\right]\\ 0<\sum_{j=1}^{\Lambda }\mu_{kj}\leq n\;\;\;\;\;\;\;\;\;\;\;\;\;\;\;\;\;\;\;\;\;\;\;\;\;\;\;\;\;\;\;\;\;\;\;,\;\;k\in \left[1,\Lambda \right] \end{array}\right. \end{array}$$

In Equation (20), $\mu_{kj}$ represents the membership degree of user $u_{j}$ to cluster $O_{k}$. The membership matrix can then be denoted by $U_{\Lambda \times n}$ and the cluster center matrix is O, which can be calculated in the following

(21) $$o_{k}=\frac{\sum_{j=1}^{n}{\left(\mu_{kj}\right)}^{2}h_{ji}}{\sum_{j=1}^{n}{\left(\mu_{kj}\right)}^{2}},k\in \left[1,\Lambda \right]$$

(22) $$\mu_{kj}=\frac{1}{\sum_{i=1}^{\Lambda }{\left\{\frac{d_{kj}}{d_{ji}}\right\}}^{2}},k\in \left[1,\Lambda \right],j\in \left[1,n\right]$$

We set the iteration times to *l* and the stop parameter to ξ, respectively. Given the user preference matrix H, SFCM randomly generates an initial membership matrix $U_{0}$ and calculates Λ initial cluster centers $o_{k},k\in \left(1,2\ldots \ldots \Lambda \right)$. According to cluster center matrix O, both cosine similarity $d_{kj}$ and membership matrix $U_{\Lambda \times n}$ can be obtained. For instance, if $d_{kj}=0$, membership degree of $u_{j}$ to $O_{k}$ is 1. Finally, the iteration is stopped, if $\left|J^{\left(l+1\right)}-J^{\left(l\right)}\right|\leq \xi$, to generate clustering results $U_{\Lambda \times k}$ and obtain cluster center matrix O. Otherwise, iterations continue to update $U_{\Lambda \times k}$ and O until reaching iteration times *l* or stop parameter ξ.

Generally, FCM constructs clusters according to the membership matrix (i.e., $O_{k}=\left\{k|\mu_{kj}=\max_{i}u_{ij},1\leq j\leq \Lambda \right\}$, $u_{j}\in O_{k}$). However, users clustered by FCM can only belong to one cluster according to her/his highest task preference, which is against the intuition that users with similar preferences for several tasks may simultaneously belong to multiple clusters. Therefore, we define clustering threshold Θ to establish these characteristic overlapping clusters. Specifically, the maximum membership value of a user in the cluster is compared with the other memberships value that he/she belongs to, then all comparison values are sorted, the largest comparison value are got among them as the threshold, which calculated by $\Theta =\underset{E_{j}}{\arg\max}{⋃}_{j=1}^{\left|U_{k}\right|}\;\;\;E_{j}$, $E_{j}={⋃}_{i=1}^{m}\min\left|\mu_{ij}^{\max}-\mu_{-ij}\right|$. The true label is obtained by the maximum average preference value among different types of tasks in a cluster; there holds

(23) $${\bar{H}}_{i}=\frac{\sum_{j=1}^{\left|U_{k}\right|}h_{j=1}}{\left|U_{k}\right|}$$

where $\left|U_{k}\right|$ is the total number of users in cluster $O_{k}$.

### 5.2. Optimization Problem Based on Lagrange Duality

#### 5.2.1. Problem Reformulation

Since the task price per meter is fixed, the income of participants can be maximized by reducing the movement distances, whereas the platform maximizes its profit by selecting participants with high service benefits. Therefore, the optimization problems in Equations (4)–(6) can be rewritten as follows

(24) $$\begin{array}{c} \max\;\;\;\;\sum_{i=1}^{m}\varphi_{i}-\sum_{j=1}^{n}C_{j}\\ s.t.\;\;\;\;\;\;\sum_{i=1}^{m}{d_{i}}_{j}\leq r_{j}^{\max},{d_{i}}_{j}>0,j=1,2\ldots \ldots n\\ \;\;\;\;\;\;\;\;\;\;\;\;\;\;\;\;\sum_{j=1}^{n}P_{i}\cdot d_{ij}\leq B_{i},i=1,2\ldots \ldots m\\ \;\;\;\;\;\;\;\;\;\;\;\;\;\;\;\;\;0<s_{ij}\leq 1 \end{array}$$

In Equation (24), $\varphi_{i}=\sum_{j=1}^{n}w_{i}\cdot \ln\left(1+s_{ij}\right)$ and $C_{j}=\sum_{i=1}^{m}\left(a_{ij}\cdot d_{ij}^{2}+b_{ij}\cdot d_{ij}+c_{ij}\right)$ represent the service benefits obtained by the platform and the costs consumed by participants, respectively.

#### 5.2.2. Lagrange Duality

Equation (24) shows that the objective function is convex with respect to $d_{ij}$ and $s_{ij}$. Hence, the Lagrange multiplier can be employed to solve this unconstrained dual problem; there holds

(25) $$\begin{array}{c} L\left(d_{ij},\alpha_{ij},{\beta_{i}}_{j}\right)=\sum_{i=1}^{m}\varphi_{i}-\sum_{j=1}^{n}C_{j}+\sum_{j=1}^{n}\alpha_{ij}\cdot \left(\sum_{i=1}^{m}d_{ij}-r_{j}^{\max}\right)+\sum_{i=1}^{m}{\beta_{i}}_{j}\cdot \left(\sum_{j=1}^{n}P_{i}\cdot d_{ij}-B_{i}\right)\\ \;\;\;\;\;\;\;\;\;\;\;\;\;\;\;\;\;\;\;\;\;\;\;\;\;\;\;\;\;\;\;\;\;\;\;\;\;\;\;\;\;\;\;\;\;\;\;\;\;\;\;\;\;\;=\sum_{i=1}^{m}\left[\varphi_{i}+{\beta_{i}}_{j}\cdot \left(\sum_{j=1}^{n}P_{i}\cdot d_{ij}-B_{i}\right)\right]+\sum_{j=1}^{n}\left[\alpha_{ij}\cdot \;\left(\sum_{i=1}^{m}d_{ij}-r_{j}^{\max}\right)-C_{j}\right] \end{array}$$

In Equation (25), the Lagrange multiplier is denoted by matrix $\beta_{m\times n}=\left[\beta_{1,*},\beta_{2,*}\ldots \ldots \beta_{m,*}\right]$, $\alpha_{n\times m}=\left[\alpha_{1,*},\alpha_{2,*}\ldots \ldots \alpha_{n,*}\right]$ and $\alpha_{ij}\geq 0$. Since the service benefit of participants is already evaluated, the dual problem can be defined by

(26) $$\begin{array}{c} \min_{\alpha_{j}\geq 0;\beta_{i}}D\left(\alpha ,\beta \right)=\min_{\alpha_{ij}\geq 0;\beta_{i}}\max_{d_{ij}>0}L\left(d_{ij},\alpha_{ij},{\beta_{i}}_{j}\right)\\ \;\;\;\;\;\;\;\;\;\;\;\;\;\;\;\;\;\;\;\;\;\;\;\;\;\;\;\;\;\;\;\;\;\;\;\;\;\;\;\;\;\;\;\;\;\;\;\;\;\;\;\;\;\;\;\;\;\;\;=\min_{\alpha_{ij}\geq 0;\beta_{i}}\max_{d_{ij}>0}\sum_{i=1}^{m}\Phi_{i}\left(\beta_{ij}\right)+\sum_{j=1}^{n}\Psi_{j}\left(\alpha_{ij}\right) \end{array}$$

In Equation (26), $\Phi_{i}\left(\beta_{ij}\right)=\;\max_{d_{ij}\geq 0}\;\;\;\varphi_{i}+\beta_{ij}\cdot \left(\sum_{j=1}^{n}p_{ij}\cdot d_{ij}-B_{i}\right)$ and $\Psi_{j}\left(\alpha_{j}\right)=\max_{d_{ij}\geq 0}\;\;\alpha_{ij}\cdot \;\left(\sum_{i=1}^{m}d_{ij}-r_{j}^{\max}\right)-C_{j}$. Because the original objective function is convex, the strong duality must satisfy the Slater condition to generate the optimal solution for this dual problem.

#### 5.2.3. Optimization Algorithm

We employ a gradient descent algorithm to iteratively solve the dual problem. The variables of the dual problem can be updated as follows

(27) $$\begin{array}{c} \langle \alpha_{ij}^{l+1},\beta_{ij}^{l+1}\rangle ={\left[\langle \alpha_{ij\partial }^{l},\beta_{ij}^{l}\rangle -\lambda \cdot \langle \frac{\partial D}{\partial \alpha_{ij}},\frac{\partial D}{\partial \beta_{ij}}\rangle \right]}^{+}\\ \;\;\;\;\;\;\;\;\;\;\;\;\;\;\;\;\;\;\;\;\;\;\;\;\;\;\;\;\;\;\;\;\;\;\;\;\;\;\;\;\;\;\;={\left[\langle \alpha_{ij}^{l},\beta_{ij}^{l}\rangle -\lambda \langle \left(d_{ij}^{l}-r_{j}^{\max}\right),\left(P_{i}\cdot d_{ij}^{l}-B_{i}\right)\rangle \right]}^{+} \end{array}$$

In Equation (27), $d_{ij}^{l}$ is the variable of the original optimization problem in the *l*th iteration, $\alpha_{ij}^{l}$ and $\beta_{ij}^{l}$ are the variables of the dual problem in the *l*th iteration, and λ is the learning step size. Participants with the best service benefits and optimal movement distances can be obtained iteratively by the platform. First, in the iteration of service benefits, participants with the best benefits in the *l*th iteration can be obtained. Then, in the gradient descent algorithm, dual variables $\alpha_{ij}^{l}$ and $\beta_{ij}^{l}$ of the *l*th iteration are obtained. Finally, $s_{ij}^{l}$, $\alpha_{ij}^{l}$ and $\beta_{ij}^{l}$ are all set for Equation (28) to generate the optimal movement distance. The iteration process does not stop until convergence conditions are met. Algorithms 1 and 2 are updated as Equations (28) and (29).

(28) $$\forall u_{j}\in U,d_{ij}^{l}=\arg\max\;\;\;\;\varphi_{i}+\beta_{ij}^{l}\cdot \left(\sum_{j=1}^{n}P_{i}\cdot d_{ij}-B_{i}\right)+\;\alpha_{ij}^{l}\cdot \;\left(\sum_{i=1}^{m}d_{ij}-r_{j}^{\max}\right)-C_{j}\;\;$$

(29) $$\forall A_{i}\in A,s_{ij}^{l}=\arg\max\varphi_{i}$$

Specifically, the complexity of Algorithm 1 is O(n), where *n* is number of task candidate. The complexity of Algorithm 2 is $O\left(m\right)$, where *m* is the number of task cluster. The complexity of overall assignment strategy is $O\left(m\times n\right)$.

When the best participants are selected for each type of task, the platform pays their task reward, updates their service benefits, and exploits scores from task publishers for the next round participant selection.

**Algorithm 1** Service benefits.
**Input:** potential participants set *U*; service benefit matrix *S*; Task set *A***Output:** the optimal participants *W* for Task $A_{i}$; Profit $f_{i}\left(s_{ij},d_{ij}\right)$
**Initialize**
W=ϕ, the number of iterations l=0Select participant $u_{0}$ randomly, $W\leftarrow u_{0}$Receive $d_{ij}^{l}$ from userCalculate the profit of each task of $u_{0}$ through Equation (5)**while**$\left|f_{i}^{l+1}\left(W\right)-f_{i}^{l}\left(W\right)\right|\leq 10\times e^{-k}$ or l≠0 **do** **if** $s_{ij}=\arg\max\varphi_{i}$ **then**
  Select participant $u_{j}$ and corresponding $s_{ij}$  Calculate the profit of the platform through Equation (5))  **return** $\langle s_{ij},f_{i}^{l}\left(s_{ij},d_{ij}\right)\rangle$  break **else**  l=l−1
  update Equation (29)  return to Line 5 **end if**
**end while**

**return** $\langle s_{ij},f_{l}\left(W\right)\rangle$



**Algorithm 2** Iterative of $d_{ij}$ progress.

**Initialization**

**for** l=0,1,2,3…… **do**
 Receive $s_{ij}^{l}$ from platform Update Equation (28) Compute the new value of $\alpha_{ij}^{l+1}$ and $\beta_{ij}^{l+1}$ using Equation (27) **if** $\left|\alpha_{ij}^{l+1}-\alpha_{ij}^{l}\right|\geq \rho$ and $\left|\beta_{ij}^{l+1}-\beta_{ij}^{l}\right|\geq \rho$, where ρ is a tunable little real number **then**  Return to Line 3 **else**  **return** $\langle d_{ij}^{l},\psi_{j}\left(d_{ij}\right)\rangle$  break **end if**
**end for**



## 6. Experiment

Gowalla, employed in this study to validate the proposed SBAMA, is a location-based real world social network dataset that allows users to share their information, including ID, access time, longitude, latitude, and location tags. The dataset collected all public check-in data between February 2009 and October 2010. There are 19,6591 nodes and 950,327 edges in Gowalla. Gowalla is mainly used to study human mobility [35]. Specifically, 500 locations and 1000 users were extracted from Gowalla as task locations and candidates, respectively. Subsequently, these 1000 task candidates were clustered into five groups, where each group maintains a task preference matrix and a corresponding service benefit matrix, containing five types of tasks. In addition, SFCM clustering algorithm and optimization algorithm in SBAMA were compared with original FCM algorithm and greedy algorithm in Dynamic Trust-Based Recruitment Framework (DTRF) [20] on MATLAB platform, respectively. Simulation parameters are given in Table 1.

### 6.1. Advantages of SFCM

The objective function iteration and clustering accuracy of SFCM were compared with those of FCM to verify the effectiveness of SFCM. Objectively, both FCM and SFCM adopt the same initial membership matrix and the simulation was repeated 100 times, where seven tests were randomly selected for observation.

**Figure 2:**
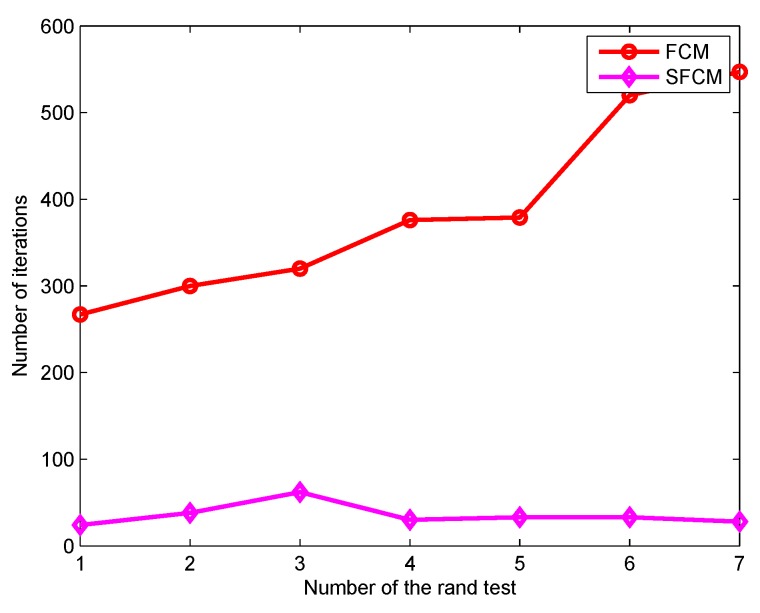
Comparison of the number of iterations.

**Figure 3:**
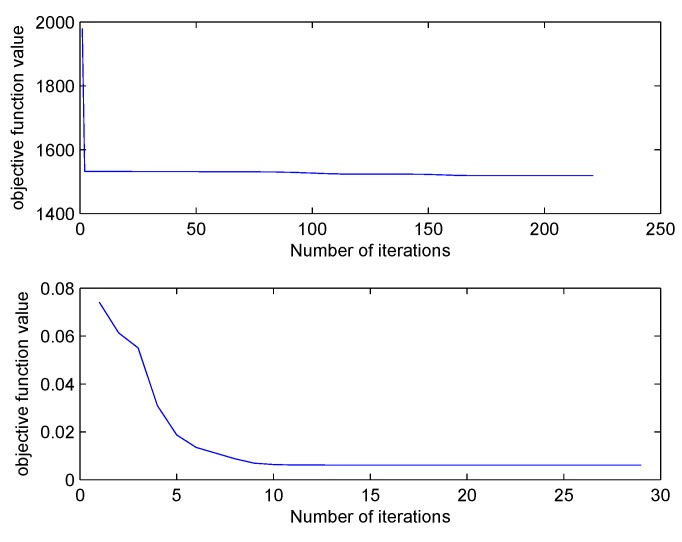
Comparison of the iterative process.

The iteration times needed by FCM and SFCM for objective function convergence are shown in Figure 2. Compared with FCM, SFCM requires a stably lower number of iterations around 30. In addition, SFCM converges quickly and has significantly short clustering time. Figure 3 illustrates the iteration of their objective function values, where the initial value of SFCM is notably much smaller than that of FCM, because the Euclidean distance in FCM is replaced by cosine similarity of SFCM to reduce the membership value.

**Figure 4:**
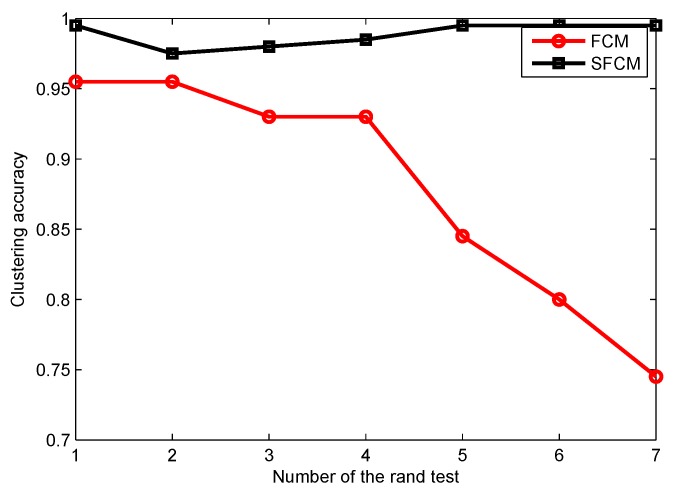
The accuracy of clustering.

**Figure 5:**
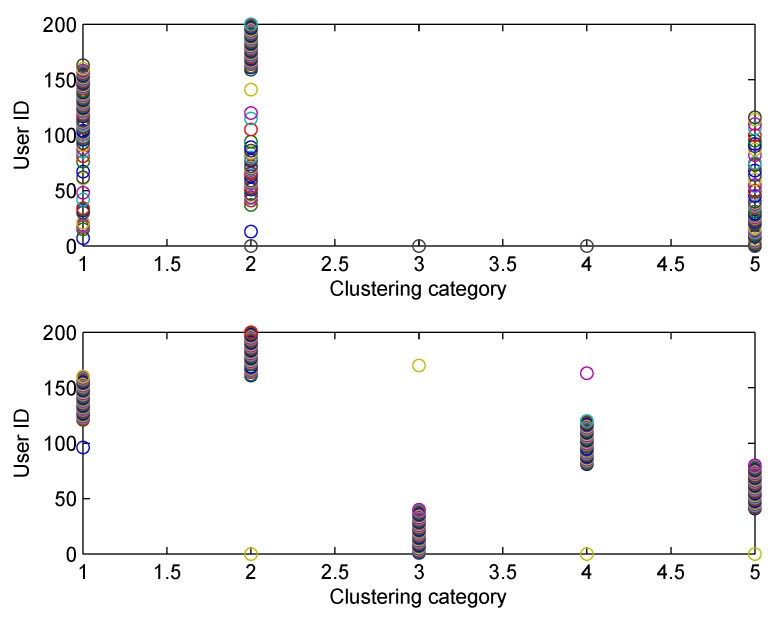
Comparison of user clustering.

**Figure 6:**
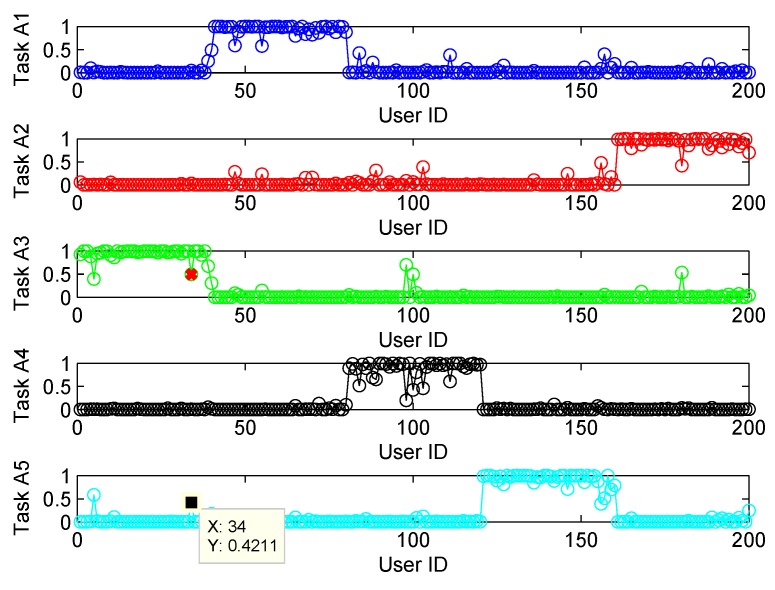
Membership matrix values of SFCM.

**Figure 7:**
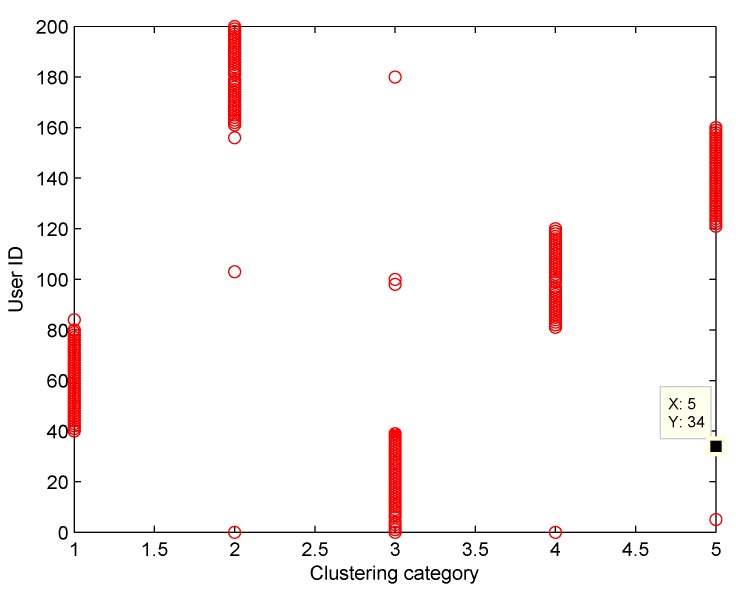
Result of SFCM cluster based on threshold.

The clustering accuracy of FCM and SFCM, given the maximum membership value, is shown in Figure 4, which is measured based on the original dataset with labels. The clustering accuracy of SFCM is generally higher than 95%, whereas the worst case of FCM is only 74.5%. Similarly, given the maximum membership value, randomly selected clustering results of FCM and SFCM are shown in Figure 5, where SFCM has a significantly better clustering result. Figure 6 shows the final membership matrix value of users from a random test. Cluster labels can be determined by Equation (22). For example, the cluster for Task A1 almost includes Users 40–80. However, according to their membership matrices, Users 34, 84, 100, 103, and 156 have similar membership values for different types of tasks. As shown in Table 2, the membership values of User 34 for Tasks A3 and A5 only differ by approximately 0.074. Besides, Users 103 and 156 have membership differences only within 0.1 for Tasks A2/A4 and Tasks A2/A5, respectively. Therefore, the task preference threshold is set to 0.1 for overlapping clustering and the clustering result of SFCM based on this threshold is shown in Figure 7. Compared with Figure 5, clusters overlap and User 34 belongs to clusters of Tassk A3 and A5 simultaneously, which is more practical for real world MCS scenarios. In short, SFCM with membership threshold can cluster users with similar task preferences, which is an effective underpinning for the subsequent optimization problem.

### 6.2. Analysis of Optimization Algorithm

**Figure 8:**
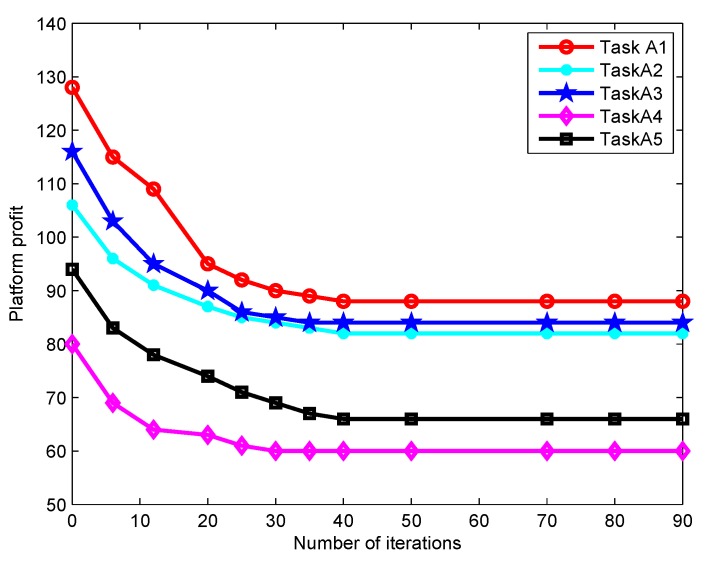
Changes in platform profit.

**Figure 9:**
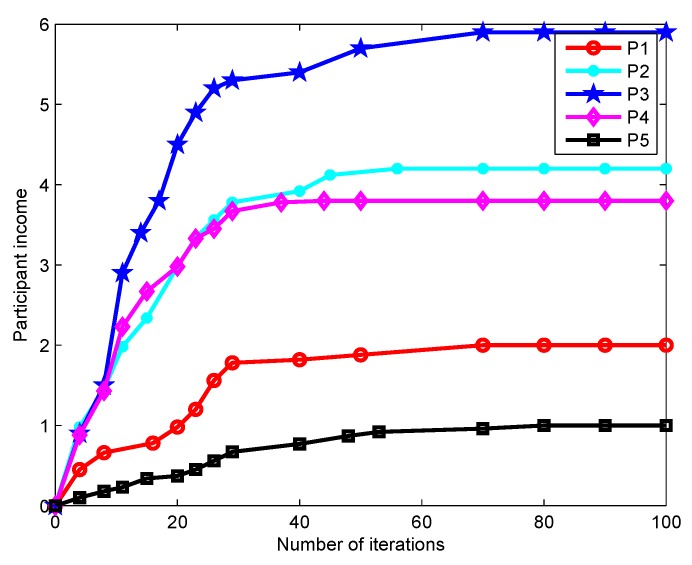
Changes in participant income.

Figure 8 shows how the platform profit gained from each type of task varies with the number of iterations. It is observed that the platform profit converges to the optimal value when the number of iterations reaches about 45, which implies the platform can stably match appropriate participants to tasks. Besides, the platform profit increases as the average service benefit increases. In Figure 9, the impact of the number of iterations on the participant income is depicted. For a fixed task price $P_{i}$, the participant income first grows sharply as the number of iterations increases and then tends to be stable. The income of Participant 3 is significantly higher than those of others. This is because his/her task is more difficult to be performed and requires a stronger sensing capacity, which thereby receives a higher payment from the platform. In Figure 8 and Figure 9, the fast iteration convergence of the proposed gradient descent algorithm for achieving the best task participants is validated.

**Figure 10:**
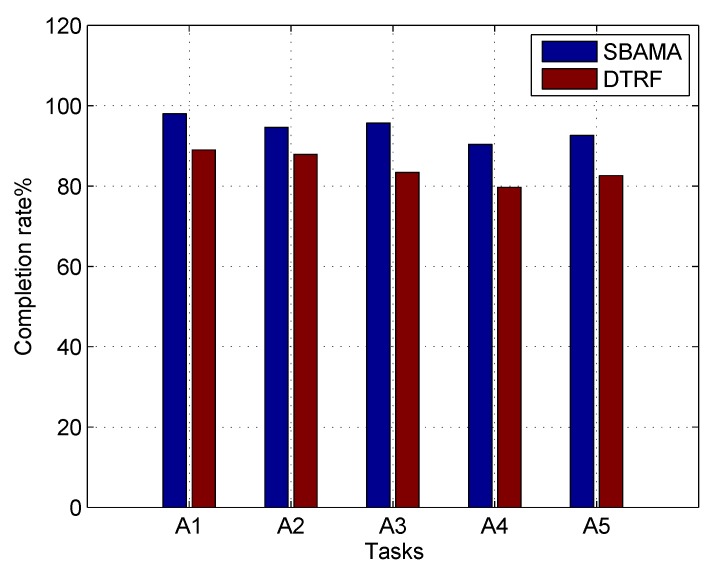
Comparison of task completion rates for different tasks.

In Figure 10, the task completion rates of DTRF and SBAMA are compared. In addition to sensing quality considered by DTRF, the proposed SBAMA also takes service benefits of participants, task preferences and real-time feedbacks from task publishers. Hence, SBAMA acquires 8% higher task completion rate.

In short, through narrowing the search range of task candidates, SBAMA effectively improves the matching accuracy of tasks assignment with fast algorithm convergence.

## 7. Conclusions

In this paper, we propose the SBAMA to quickly and accurately match MCS tasks with the most appropriate participants to improve the task completion rate and data credibility. Firstly, the service benefit of participants is modeled based on their task difficulty, task history, sensing capacity and sensing positivity to improve the accuracy of task assignment. Then, task candidates are clustered according to their task preference to narrow the search range. Finally, the gradient descent algorithm is designed to select the optimal participants in each cluster. Simulation results verify that the proposed SBAMA can quickly find the most appropriate participants to meet the requirements of multiple concurrent types of tasks under a massive user scenario, for example, crowded road condition monitoring. Although the proposed SBAMA can be applied to the scenario of massive users and numerous concurrent tasks, the strategy still has some limitations for the scenario where participants are sparse, which can lead to a low matching accuracy between the task and the participant. In the future, we will focus on the sparse participant scenario and study the associated task assignment strategy.

## Figures and Tables

**Table 1:** Parameter settings.

Parameters	Value
target region ($km^{2}$)	600×50
types of task	5
number of unit tasks for each task	$\left[20,200\right]$
participant service benefit value	$\left(0,1\right)$
user preference value	$\left(0,1\right)$
$a_{ij}$	$\left(0,1\right)$
$b_{ij}$	$\left(0,1\right)$
$c_{ij}$	$\left(1,2\right)$
inherent sensing cost $c_{\mathrm{ij}}$	(1,4)
maximum movement distance $r_{j}^{\max}$(km)	10
task effective time (min)	$\left[20,60\right]$
task price per meter $p_{ij}$($/m)	$\left(0.2,0.5\right)$
number of candidate users	200
ξ	$10^{-5}$
$w_{i}$	$\left(100,300\right)$
$B_{i}$	$\left[2000,5000\right]$

**Table 2:** Similar membership values of randomly tested users.

Task User	34	84	100	103	156
A1	0.046355202	0.423835317	0.0043460630	0.021614770	0.087140617
A2	0.034177025	0.046220128	0.0661477153	0.388445033	0.479426570
A3	0.494624410	0.003243961	0.4977484533	0.009119740	0.002899209
A4	0.003762370	0.517018454	0.4254306807	0.458955766	0.039708579
A5	0.421080991	0.009682137	0.0063270875	0.121864688	0.390825023
